# Isolation and identification of the broad-spectrum high-efficiency phage vB_SalP_LDW16 and its therapeutic application in chickens

**DOI:** 10.1186/s12917-022-03490-3

**Published:** 2022-11-03

**Authors:** Shengliang Cao, Wenwen Yang, Xihui Zhu, Cheng Liu, Jianbiao Lu, Zhenshu Si, Lanying Pei, Leilei Zhang, Wensi Hu, Yanlan Li, Zhiwei Wang, Zheyu Pang, Xijuan Xue, Yubao Li

**Affiliations:** 1 Phage Research Center, Liaocheng University, No. 1 Hunan Road, 252000 Liaocheng, Shandong China; 2 School of Agricultural Science and Engineering, Liaocheng University, No. 1 Hunan Road, 252000 Liaocheng, Shandong China; 3 Shandong Sinder Technology Co., Ltd., Sinder Industrial Park, Shungeng Road, Zhucheng Development Zone, Weifang, Shandong 262200 China

**Keywords:** *Salmonella*, Phages vB_SalP_LDW16, Biological characteristics, Recovery rate, Phage therapy

## Abstract

**Background:**

*Salmonella* infection in livestock and poultry causes salmonellosis, and is mainly treated using antibiotics. However, the misuse use of antibiotics often triggers the emergence of multi-drug-resistant *Salmonella* strains. Currently, *Salmonella* phages is safe and effective against *Salmonella*, serving as the best drug of choice. This study involved 16 *Salmonella* bacteriophages separated and purified from the sewage and the feces of the broiler farm. A phage, vB_SalP_LDW16, was selected based on the phage host range test. The phage vB_SalP_LDW16 was characterized by the double-layer plate method and transmission electron microscopy. Furthermore, the clinical therapeutic effect of phage vB_SalP_LDW16 was verified by using the pathogenic *Salmonella* Enteritidis in the SPF chicken model.

**Results:**

The phage vB_SalP_LDW16 with a wide host range was identified to the family *Siphoviridae* and the order *Caudoviridae*, possess a double-stranded DNA and can lyse 88% (22/25) of *Salmonella* strains stored in the laboratory. Analysis of the biological characteristics, in addition, revealed the optimal multiplicity of infection (MOI) of vB_SalP_LDW16 to be 0.01 and the phage titer to be up to 3 × 10^14^ PFU/mL. Meanwhile, the phage vB_SalP_LDW16 was found to have some temperature tolerance, while the titer decreases rapidly above 60 ℃, and a wide pH (i.e., 5–12) range as well as relative stability in pH tolerance. The latent period of phage was 10 min, the burst period was 60 min, and the burst size was 110 PFU/cell. Furthermore, gastric juice was also found to highly influence the activity of the phage. The clinical treatment experiments showed that phage vB_SalP_LDW16 was able to significantly reduce the bacterial load in the blood through phage treatment, thereby improving the pathological changes in the intestinal, liver, and heart damage, and promoting the growth and development of the chicken.

**Conclusions:**

The phage vB_SalP_LDW16 is a highly lytic phage with a wide host range, which can be potentially used for preventing and treating chicken salmonellosis, as an alternative or complementary antibiotic treatment in livestock farming.

## Introduction

Salmonellosis is a zoonosis caused by *Salmonella* and has more than 2600 serotypes [1]. Most of the *Salmonella* serotypes are pathogenic to people, pigs, and chickens [2], and transmit to different animals in various ways [3]. Poultry is critical for transmitting *Salmonella* with different pathogenicity [4]. Chicken salmonellosis is a chronic or acute infectious disease caused by multiple *Salmonella* serotypes. The most prominent *Salmonella* serotypes isolated from chicken in China include the *Salmonella* Enteritidis (SE), *Salmonella* Pullorum (SP), and *Salmonella* Typhimurium (ST), which cause diarrhea, a decline in reproductive performance, and high mortalities [1]. *Salmonella* is prevalent in farms and can be vertically transmitted severely affecting the functioning of the poultry farms and hatcheries.

The present treatment for chicken salmonellosis relies mostly on antibiotic treatment. The long-term, large-dose, and misuse use of antibiotics lead to the emergence of the multidrug-resistant *Salmonella*, destruction of the balance of the intestinal microbiota, reduction of the immune function, and animal poisoning caused by the excessive antibiotic doses, antibiotic drug residues in animal products and destruction of the environmental ecosystem [5, 6]. Therefore, there is an urgent need for developing a safe and efficient antibiotic substitute with minimal toxicity to the host [7]. Phage is a highly efficient and specific virus infecting the bacteria as a natural antibacterial agent in the following ways. Phages target specific multidrug-resistant bacteria in phage therapy but do not affect the intestinal microorganisms [8, 9]. Moreover, the phages infect the bacteria without modifying the intestinal flora and can reproduce themselves by infecting the host bacteria, reducing the use of medications. Phages exert a bactericidal mechanism completely different from that of antibiotics. Phages are not limited by microbial antibiotic resistance; have a short research cycle and low cost, and are easy to update. Therefore, several studies have also focused on using bacteriophages for controlling *Salmonella* in chickens.

The study aimed to identify the biological characteristics and therapeutic effect of the newly isolated chicken *Salmonella* phage vB_SalP_LDW16, for providing a theoretical basis for preventing and controlling the *Salmonella* disease in chickens. In this study, 16 *Salmonella* phages were isolated and purified from the sewage and feces of the broiler farm, to identify a new phage vB_SalP_LDW16 having a wide host range that can cleave 88% (22/25) of different serotypes of *Salmonella* and protect the chickens infected with the pathogenic *Salmonella* Enteritidis.

## Result

### Isolation, purification and host range determination of the *Salmonella* phage vB_SalP_LDW16

**Fig. 1:**
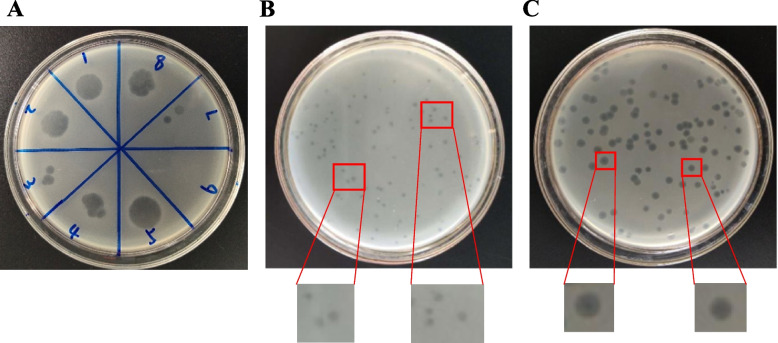
Isolation and purification of the *Salmonella* phage vB_SalP_LDW16. **A** Sample filtrate displays the clear phage plaques on the double-layer plate overlays with suspensions of *Salmonella* after incubation for 24 h, depicting lysed bacteria, 1–8 represent partial filtrate samples. **B** The phage vB_SalP_LDW16 crude filtrate. After 24 h of incubation, clear and different sized plaques were displayed on the *Salmonella* lawn. **C** The purified phage vB_SalP_LDW16 displays clear and uniform patched plaques on a *Salmonella* lawn after incubation for 24 h

Bacteriophages are the most diverse organisms on earth and exist in the soil, water, air, ocean, drinking water, food, and other environments [10]. In this study, 16 samples were isolated from the poultry farm sewage and feces in the Shandong province, China. To verify whether these samples had phages, the filtrate of these samples was visualized using Double-layer plate drip method and the results showed that all these samples contained *Salmonella* phage and different plaques morphologies (Fig. 1-A, Partial results are shown). Then, these phages were named and their specific information has been shown in Table 1. To verify the host range, the isolated phages were tested with 25 strains of *Salmonella* of different serotypes stored in the laboratory. The results indicated the lytic effect of the phage vB_SalP_LDW16 on the 25 *Salmonella* serotypes to be 88%. The lytic effect of vB_Sal_LDW4, vB_Sal_LDW13, and vB_Sal_LDW15 was found to be 60%, which is the lowest. The lytic effect of the remaining phages was above 72%. The same phage was found to lyse the different serotypes of *Salmonella*, including *S.* Pullorum, *S.* Enteritidis*,* and *S.* Typhimurium. This indicated that there is no correspondence between the *Salmonella* phage and *Salmonella* serotypes. The novel phage vB_SalP_LDW16 was found to have high lytic potential against *S.* Pullorum, *S.* Enteritidis*,* and *S.* Typhimurium and had a wide range of hosts. Hence, the phage vB_SalP_LDW16 was used for further investigations. To study the biological function of the phage vB_SalP_LDW16, the phage was first purified by the double-layer agar plate method, and the results are shown in Fig. 1-B that phage plaques of different sizes and shapes were displayed on the plate. After 6 cycles of purification, the vB_SalP_LDW16 phage plaques of uniform size and shape were observed on the plate (Fig. 1-C) and these results indicated a new type of *Salmonella* phage with broad-spectrum and high lytic activity was successfully isolated and purified.

**Table 1:** Lysis rate of the isolated bacteriophages

Phage	Number of lysed strains^a^			lysis rate (%)
	SE	SP	ST	
vB_SalP_LDW1	6	8	7	84
vB_SalP_LDF2	6	6	7	76
vB_SalP_LDF3	5	6	7	72
vB_SalP_LDW4	4	4	7	60
vB_SalP_LDW5	6	8	7	84
vB_SalP_LDD6	6	8	5	76
vB_SalP_LDW7	5	8	7	80
vB_SalP_LDW8	5	8	7	80
vB_SalP_LDF9	6	8	7	84
vB_SalP_LDD10	6	8	6	80
vB_SalP_LDF11	6	8	7	84
vB_SalP_LDW12	5	8	7	80
vB_SalP_LDW13	6	6	3	60
vB_SalP_LDF14	7	5	6	72
vB_SalP_LDW15	6	6	3	60
vB_SalP_LDW16	7	8	7	88

^a^ *SE* stands for *Salmonella* Enterica, *ST* stands for *Salmonella* Typhimurium, *SP* stands for *Salmonella* Pullorum

### Micrograph and categorization of the phage vB_SalP_LDW16

**Fig. 2:**
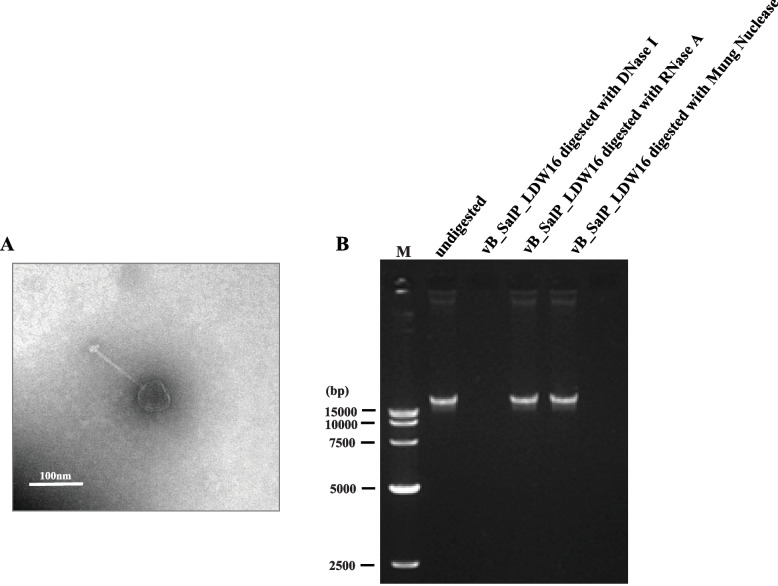
Micrograph and categorization of the phage vB_SalP_LDW16. **A** The transmission electron micrograph of the phage vB_SalP_LDW16 virus particles was negatively stained with 2% uranyl acetate. The scale bar represents 100 nm. **B** The agarose gel electrophoresis analysis of the nucleic acid of the phage vB_SalP_LDW16 after restriction endonuclease digestion. Lane 1, M, 15 kb DNA ladder; Lane 2, vB_SalP_LDW16, undigested; Lane 3, vB_SalP_LDW16 digested with DNase Ι; Lane 4, vB_SalP_LDW16 digested with RNase A; Lane 5, vB_SalP_LDW16 digested with Mung Nuclease

To classify the phage vB_SalP_LDW16 into the morphotype-specific groups, the morphology of the phage was analyzed by Transmission electron microscopy (TEM). Firstly, the phage vB_SalP_LDW16 lysate having a titer of 3 × 10^14^ PFU/mL was produced by plate amplification. The TEM images of the phage vB_SalP_LDW16 are shown in Fig. 2-A. Structurally, the phage vB_SalP_LDW16 has an icosahedral head of approximately 55 ± 3 nm diameter and long, non-contractile tails of about 115 ± 3 nm length (Fig. 2-A). According to the guidelines of the International Committee on Taxonomy of Viruses, the morphological characteristics of the phage vB_SalP_LDW16 suggested it belongs to the *Siphoviridae* family of the *Caudovirales* order [11–13]. To determine the genetic type of the isolated phage [14, 15], the nucleic acid was isolated from the particles of phages and digested using the restriction enzymes. The agarose gel electrophoresis showed the nucleic acid of the phage vB_SalP_LDW16 to be sensitive to DNase I, but was not sensitive to the RNase A and Mung Nuclease, indicating the genomic material to be a double-stranded DNA (Fig. 2-B).

### Biological characteristics of the phage vB_SalP_LDW16

**Fig. 3:**
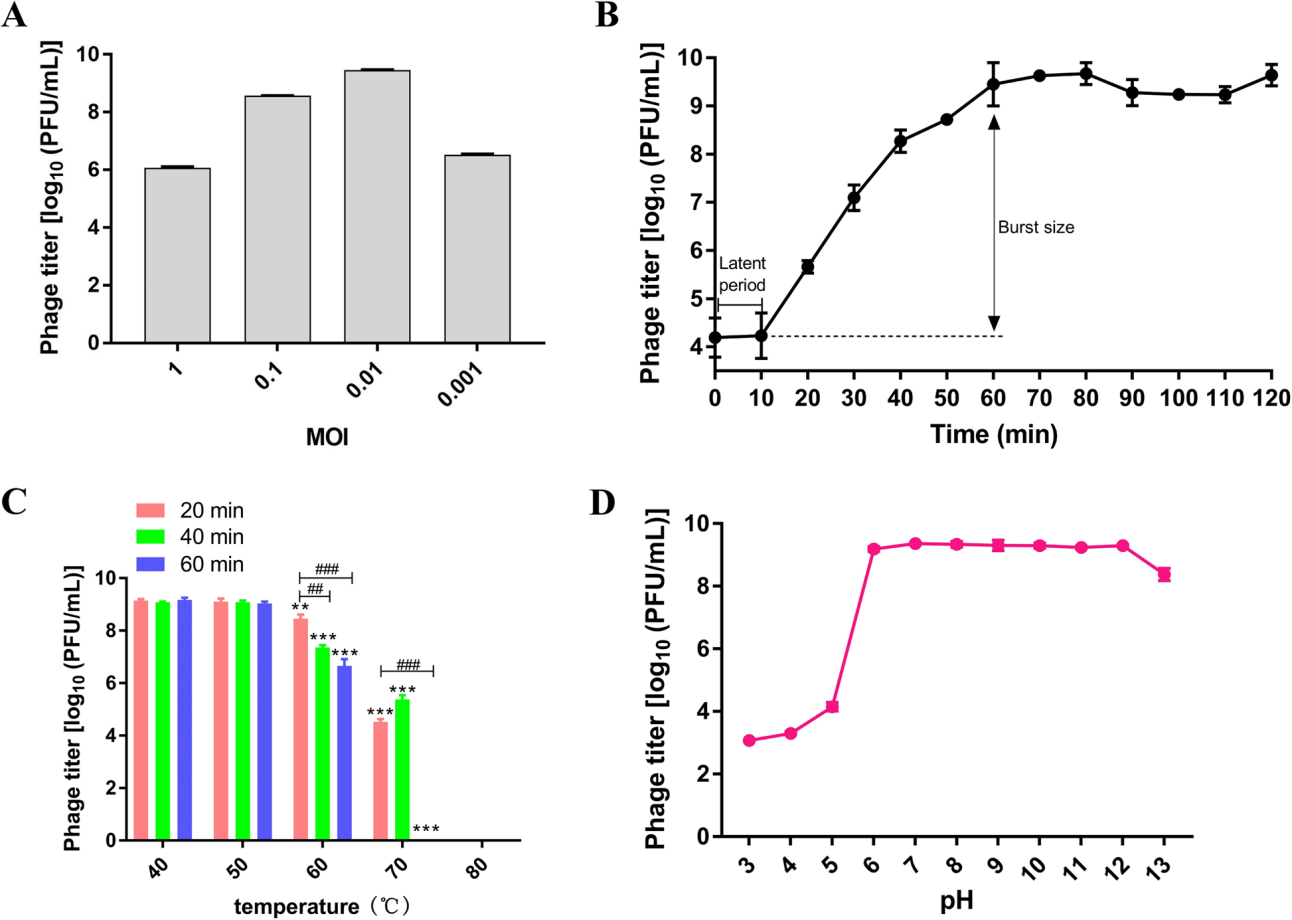
Biological characteristics of the phage vB_SalP_LDW16. **A** MOI of the phage vB_SalP_LDW16. Phage titers were measured at different MOIs. **B** One-step growth curve of the phage vB_SalP_LDW16. The titer of the phage vB_SalP_LDW16 was determined every 10 min. **C** The thermal stability of the phage vB_SalP_LDW16. The phages were cultured in a water bath at different temperatures. **p < 0.01, ***p < 0.001 versus samples at 40 ℃ under the same incubation time, ^###^*p* < 0.001 versus sample at 20 min at the same incubation temperature. **D** The pH stability of the phage vB_SalP_LDW16. The phages were incubated in different acid–base environments for 1 h. **A**, **B**, **C**, **D** The phage titers were determined using the double-layer agar method and each data point represented the mean values ± standard deviations (SD) from at least three replicate experiments

To determine the optimal multiplicity of infection (MOI) of the phage vB_SalP_LDW16, the dilutions of *Salmonella* in the logarithmic growth phase were infected with different amounts of phage vB_SalP_LDW16, and the titer of the phage was measured after 3 h incubation. The results indicated the titer of the bacteriophage to be 1.2 × 10^6^ PFU/mL, 3.7 × 10^8^ PFU/mL, and 3.4 × 10^6^ PFU/mL, when the MOI was 1, 0.1, and 0.001, respectively. When MOI was 0.01, the phage vB_SalP_LDW16 was found to generate a maximum titer of 2.8 × 10^9^ PFU/mL (Fig. 3-A). Therefore, the optimal MOI of the phage vB_SalP_LDW16 infected with *Salmonella* was considered to be 0.01. As evident in Fig. 3-B, a one-step growth curve analysis of the phage vB_SalP_LDW16 revealed almost no change in the latent period of the first 10 min, with a rapid increase within 10 to 60 min, and reached a stable period after 70 min. Furthermore, the average burst size of the phage vB_SalP_LDW16 was estimated as 110 PFUs/infected cells.

To investigate the viability of the phage vB_SalP_LDW16 under diverse environmental conditions, the thermal and pH stabilities of the phage were estimated by determining the changes in the survival based on the number of plaque-forming units (PFU). As shown in Fig. 3-C, vB_SalP_LDW16 was found to retain high levels of infectivity following incubation in water at 40 ℃ or 50 ℃ for 20 min, 40 min, and 60 min respectively, but was sensitive to the higher temperatures. The stability of the phage was gradually decreased at a temperature exceeding 60 ℃, and the activity of the phage gradually decreased with increasing incubation time at the same temperature. However, the bacteriophages could not be detected after incubation at 70 ℃ or 80 ℃ for 1 h. In addition, the stability of the isolated and purified phage vB_SalP_LDW16 was measured at pH 2–13. The experimental data revealed the phage vB_SalP_LDW16 to maintain good activity under the condition of pH 6–12, but the activity of the phage vB_SalP_LDW16 was found to decrease significantly at pH 2–5 and pH 13. These results indicated that the phage vB_SalP_LDW16 had better stability under alkaline conditions, but was sensitive to the acid or strong base (Fig. 3-D).

### Recovery rate of the phage vB_SalP_LDW16

**Fig. 4:**
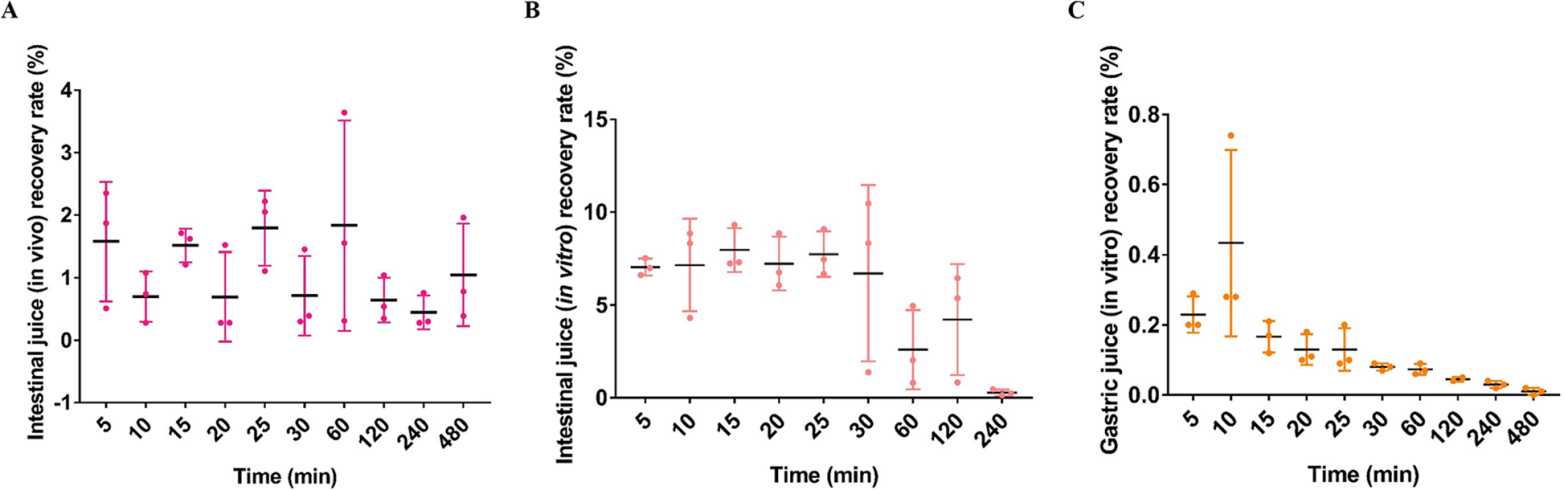
Recovery rate of the phage vB_SalP_LDW16. **A** The intestinal juice recovery rate of the feeding phage in the chickens *in vivo*; **B** Intestinal juice recovery rate of phage in the chickens *in vitro*; **C** Gastric juice recovery rate of phage in the chickens *in vitro*. **A**, **B**, and **C** The phage titers were determined using the double-layer agar method. Each data points represent the mean values ± standard deviations (SD) from at least three replicate experiments

Bacteriophage is a natural agent that removes bacteria. Many researchers have sometimes found the oral phage to be less effective in treating *Salmonella* [16, 17]. To study the effects of chicken gastric juice and intestinal juice on the activity of the phage vB_SalP_LDW16, the lysis activity and titer of the phage were tested in vitro and in vivo. In both the in vitro and in vivo experiments, the phage content of the control group was found to be zero (data not shown). Figure 4-A shows that in the in vivo experiment, the phage recovery rate of the phage feeding group was lower (about 1.5%), and there was no significant difference (*P* > 0.05) between the different times. Similarly, in the in vitro experiments, the recovery rate of the phages in the intestinal fluid of the phage mixture group was found to be slightly higher (about 6%) within 5–30 min and significantly decreased after 30 min (Fig. 4-B), while the recovery rate of the phage in the gastric juice was only about 0.2% (Fig. 4-C). The above results indicated that there was a severe loss in the activity of the phage vB_SalP_LDW16 after passing through the stomach, which was also consistent with the alkali- and acid-intolerant characteristics of the phage vB_SalP_LDW16.

### The phage vB_SalP_LDW16 rescued chickens from the *Salmonella* infection

**Fig. 5:**
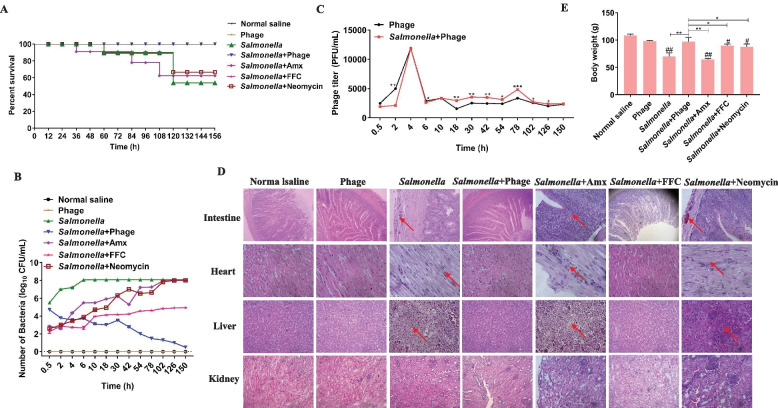
Therapeutic effect of the phage vB_SalP_LDW16 on the chicken model infected with *Salmonella*. **A** Survival rates. The chickens were intraperitoneally injected with 10^8^ CFU/mL of *S.* Enteritidis S64. Two hours later, the chickens were orally fed with 10^6^ PFU/mL of phage, amoxicillin (2.5 mg/mL), florfenicol (2 mg/mL), and neomycin (100 mg/mL). Chickens injected with bacteria, phage or saline only were set as a control group; **B** Dynamic changes in the bacteria in the blood in different groups. **C** Dynamic changes in the phage in the feces in different groups. **D** Histopathological images of the representative tissues and organs. The red arrow represents the lesion area. Intestine (HE, 100 ×), Heart (HE, 400 ×), Liver (HE, 400 ×), Kidney (HE, 400 ×). **E** The average weight of the different groups at the end of the experiment. The error bars indicate the standard deviations. Asterisks and pound signs indicate a statistically significant difference (*P* < 0.05), in which “#” (#*p* < 0.05, ##*p* < 0.01) are compared with the blank control group and “*” (*p < 0.05, **p < 0.01) are compared with the phage-treated group

To corroborate whether the phages can prevent the proliferation of *Salmonella* in vivo, the infection and treatment experiments were performed in chickens. First, the LD50 of *Salmonella* Enteritidis S64 was determined to be 10^8^ CFU/mL (data not shown), and the survival rate of chickens in the control group of *Salmonella* Enteritidis S64 was identified as 50% (Fig. 5-A). In the chicken treatment experiment, the chickens in each group were administered antibiotics or phages orally for 3 consecutive days. The survival rate of the phage treatment group one week later was 100%, while the survival rates of the florfenicol, amoxicillin, and neomycin treatment groups after one week were 100%, 65%, and 60%, respectively (Fig. 5-A). The survival rate of chickens in the phage control group and the saline control group was 100%, also indicating that the phage vB_SalP_LDW16 had no side effects on the chickens (Fig. 5-A). In addition, the dynamics of bacteria were determined in the blood of each group of chickens. The *Salmonella* control group showed that 6 h after inoculation, the bacteria reached a peak in the blood. The bacteria load in the phage treatment group was found to gradually decrease, and the bacteria were almost eliminated after 78 h. However, the number of bacteria in the amoxicillin and neomycin treated groups was low in the first 4 h after administration, and gradually increased after 4 h until the bacterial peak was attained. The number of bacteria in the florfenicol treated group remained low (Fig. 5-B). At the same time, dynamic changes were measured in the phages in feces. The results are shown in Fig. 5-C indicating that the number of bacteriophages in the feces could be detected in a short time, peaked at 4 h after administration, and then remained at a certain level. The phage content of the phage-treated group was also found to be higher than that of the phage group 4 h post-dose.

Additionally, the tissues of the intestine, liver, kidney, and heart of the dead chickens in each group or at the end of the experiment each group were grossly examined. The histological analysis indicated that compared to the blank control group, all the tissues and organs in the phage treatment group and the phage control group could be developed normally. However, in the *Salmonella* control group, amoxicillin treatment group and neomycin treatment group, the intestinal cells were necrotic and mucosal lamina propria hemorrhage comprised mainly of macrophages; there were a large number of necrotic foci of different sizes in the liver, and the liver cells in the necrotic foci were necrotic and disintegrated; there was no obvious lesion in the kidney and there were no related pathological changes upon treatment with florfenicol. At the end of the experiment, the weight of chickens was measured in each group. The results are shown in Fig. 5-E, compared to the blank control group, the weight of chickens decreased significantly (*P* < 0.05) in the *Salmonella* control group and amoxicillin, florfenicol, and neomycin treatment group while there was no significant change in the weight of the chickens in the phage treatment group and phage control group. At the same time, the weight of chickens in the phage treatment group was found to be significantly higher than that in the other treatment groups. These bodyweight indices were positively correlated with the number of bacteria in each group. In conclusion, the phage treatment experiments showed that the phage vB_SalP_LDW16 could fully protect the chickens from *Salmonella* infection, improving the pathological changes in the intestinal, liver, and heart damage, and promoting the growth and development of the chickens.

## Discussion

Bacteriophages mainly abound in environments like sewage, feces, and soil, and constitute the most abundant organisms on earth [18]. In the early twentieth century, the phages were proposed for treating bacterial diseases in humans and animals [19]. In 1940, phage therapy received a setback with the advent and use of antibiotics and was shifted to basic research [20, 21]. However, the emergence of a large number of multidrug-resistant bacteria in recent years due to the unprecedented use of antibiotics resulted in increased attention towards phage therapy since it has proven to have a good therapeutic effect in treating multidrug-resistant bacteria. The phage was applied for treating bacterial diseases such as in humans, animals, and plants, with a remarkable effect on the multi-drug resistant bacteria.

*Salmonella*-killing bacteriophages exist in the natural environment and can be isolated from sewage and poultry litter [22]. In our study, 16 phages were isolated from the poultry farm such that their separation ratios in the sewage, feces and litter were respectively 56.25%, 31.25%, and 12.5%, as previously described, and the separation ratio was the highest in sewage [23]. Generally, bacteriophages have strong host specificity, however, this serves as a limiting factor for treating bacterial infections [24]. Therefore, the 16 bacteriophages isolated were verified with 25 different subtypes of *Salmonella* stored in the laboratory, and the results showed that only 3 out of the 16 phages were sensitive to the two serotypes of *Salmonella* (*S.* Enteritidis and *S.* Typhimurium). The remaining 13 phages were found to be sensitive to the three serotypes of *Salmonella* (*S.* Enteritidis, *S.* Typhimurium, and *S.* Pullorum), indicating that the host ranges of the phages were not related to the serotype. Previous studies have reported that the phage vB_SalP_TR2 successfully infects *S.* Albany, *S.* Corvallis, *S.* Newport, *S.* Kottbus and *S.* Istanbul [15]. In addition, studies have reported that Salmacey3, a *Salmonella* phage, can infect *E. coli* and have a lytic effect on *S.* typhi and Citrobacter freundii [1], indicating that the phage can not only infect the cross-species but also cross-genus. Among the 16 isolated phages, the vB_SalP_LDW16 was found to be a novel phage with high lytic potential against *S.* Pullorum, *S.* Enteritidis, and *S.* Typhimurium respectively and was found to possess a wide range of hosts. Hence, the phage vB_SalP_LDW16 was used for further investigations. Next, the morphology of the phage vB_SalP_LDW16 was observed through an electron microscope, and the results showed the bacteriophage to have an icosahedral head and long, non-contractile tails. According to the International Classification of Virology [11–13], the phage vB_SalP_LDW16 belonged to the family *Siphoviridae*, order *Caudoviridae*, and the phage vB_SalP_LDW16 was found to have a double-stranded DNA based on the characteristics of phage digestion by restriction enzymes.

The biological characteristics of the phage vB_SalP_LDW16 having a relatively broad host range were investigated. The phage vB_SalP_LDW16 lysate with a titer of 3 × 10^14^ PFU/mL was obtained by plate amplification and was found to be higher than most *Salmonella* phage titer [25, 26]. The resistance to heat and pH of bacteriophages was found to be essential for biocontrol applications, hence relevant performance was determined under the conditions of phage vB_SalP_LDW16 at an optimal MOI of 0.01. The phage vB_SalP_LDW16 was found to be stable during 40–60 °C and decreased when the temperature was above 60 °C. Then, the phage vB_SalP_LDW16 was found to exhibit relatively high thermal stability [27, 28]. However, the phage vB_SalP_LDW16 titer was found to be significantly reduced when the pH < 5, and when the pH 2 was reached, the phage was inactivated. This result is consistent with most studies suggesting that the phages are more resistant to alkali than acid [29, 30]. The latent period of the phage mainly referred to the time from the phage adsorption to host bacteria for the lysis and the release of progeny phage, while the burst size referred to the number of progeny phage released by single host bacteria, and the incubation period and burst size of the phage was also the main indicators of the ability to lyse bacteria [14]. This indicated that the shorter the incubation period, the stronger would be the ability to lyse bacteria [31]. According to related reports, the latent period and burst size of the *Salmonella* phage vB_SalP_TR2 were 15 min and 211 PFU/cell, respectively [15]; the latent period of *Salmonella* phages Salmacey1 and Salmacey2 was about 30–40 min, and the burst size was 80–90 PFU/cell [1]. In the study, the latent period of the phage vB_SalP_LDW16 was found to be only 10 min and the burst size was 115 PFU/cell which showed that the latent period of phage vB_SalP_LDW16 was shorter than that (25–65 min) of many other reported *Salmonella* phages [32–34]. This indicated that the phage vB_SalP_LDW16 could efficiently lyse the host bacteria.

Bacteriophage is a natural agent for removing bacteria. However, researchers have found the efficacy of oral phage in treating *Salmonella* to be poor [16, 17]. The main reason is because of the high acidity of the gastric juice which inactivates the phage in the stomach, weakening the efficacy of the phage. However, in this study, the recovery rate of the phage vB_SalP_LDW16 in the intestinal juice in vitro and in vivo was found to be about 1.5% and 6%, respectively, while the recovery rate of the gastric juice was only 0.2%. This indicated that the phage vB_SalP_LDW16 was probably easily affected by the acidic environment of the gastric juice consistently supporting its biological characteristics of intolerance to the acidic environment. Ma et al. showed that microencapsulation technology effectively protects the oral phages from gastric acid and bile, and enhances the antibacterial effect of the phages [35]. Gene editing is a hot spot in the current life science research, and CRISPR/Cas is an important tool for gene-editing capable of modifying related biological genes, through knockout, insertion, and point mutation [36]. Adding a gene element for antibacterial resistance to the phage genome has been reported to solve the problem of bacteria developing resistance to the phage [37]. Artificially modifying the phage tail protein genes can replace or expand the host spectrum [38]. Therefore, to increase the acid resistance of the phages, the acid-resistant genes can be added through gene-editing technology to improve the gastric overpass rate of the phages, serving as one of the key technologies for solving the low gastric overpass rate of the oral phages in livestock and poultry clinic in the future.

To date, phage therapy has shown great potential for bacterial diseases such as *Salmonella* [39] and *Campylobacter* [40] in poultry. In this study, the efficacy of our isolated phage was verified by studying the therapeutic effect of the phage vB_SalP_LDW16 on the *Salmonella* infection in chickens. Amoxicillin, florfenicol, and neomycin, which are commonly used in treating poultry *Salmonella*, were selected as the therapeutic control in the treatment experiments. In the experimental results, the phage-treated chickens infected with *Salmonella* Enteritidis were found to have a survival rate of 100%, while the amoxicillin and neomycin-treated chickens were found to have a survival rate of about 40%. The dynamic determination of bacteria in the blood of each group showed the bacteria counts to decrease significantly in the first 4 h after amoxicillin and neomycin treatment, and increase rapidly to the peak after 4 h, while the number of bacteria was found to decrease gradually in the phage-treated group, and the bacteria were almost cleared after 78 h. At the same time, upon histopathological observation of each group, the degree of the pathogenesis of the heart, liver, and intestine in the amoxicillin and neomycin treatment group was found to be significantly higher than that in the phage-treated group. Compared to the other treatment groups, phage therapy was found to promote the growth and development of the clusters. *Salmonella* Enteritidis and *Salmonella* Typhimurium were reported as the most common serotypes, showing the highest incidence of resistance to polymyxin (100%), followed by ampicillin (68.7%), and the isolation rate of the multidrug-resistant *Salmonella* was 53.7% [41]. In the commercial broiler supply chain, the *Salmonella* species isolates are resistant to at least one of the antibiotics, such as doxycycline (94.34%), or neomycin (33.02%) [42]. A QRDR point mutation in the gyrA gene of the South Korean non-typhoid *Salmonella* tends to reduce the sensitivity to fluoroquinolones, resulting in drug resistance [43]. As with antibiotic therapy, the specificity of the phage to the strain might account for the high success rate and safety of the phage therapy [44]. In this study, although the recovery rate of the phage vB_SalP_LDW16 was low, the phage in the phage control group and phage treatment group could be detected in a short time, and the phage treatment group was found to promote the growth and development of chickens compared to the other treatment groups. This also showed that the low-dose phage has a good therapeutic role. Phage therapy is a promising method for combatting the rise of multidrug-resistant bacteria. At present, bacteriophages are paired with antibiotics, to improve efficacy when used clinically [45]. The above results also showed that the phage vB_SalP_LDW16 can fully protect the chickens from *Salmonella* infection and improve the pathological changes in the intestinal, liver, and heart injury.

## Conclusions

This study isolated 16 *Salmonella* phages, and these phages were found to lyase two or more serotypes of *Salmonella*, and phage vB_SalP_LDW16 having a broad host range is capable of lysing 88% of *Salmonella* strains of our laboratory. Meanwhile, the bacteriophage vB_SalP_LDW16 was found to act as a highly effective antimicrobial agent for controlling avian salmonellosis due to its short incubation period, large burst size, and stability to a wide range of pH and temperature. But, the gastric juice was found to have a greater influence on the activity of the phage, so protective agents should be added during the actual production in the future.

## Materials and methods

### Bacterial strains

The *Salmonella* strains used in this study were obtained from the Phage Research Center, Liaocheng University, and these strains were isolated from the livers of chickens with clinical signs of salmonellosis collected from the farms in the Shandong Province. The *Salmonella* strains in this paper were isolated in recent years, with a total of 25 strains, mainly including three serotypes of *Salmonella* Enteritidis, *Salmonella* Pullorum and *Salmonella* Typhimurium, and the specific information is enlisted in Table 2. All the bacteria were cultivated in Luria–Bertani (LB) broth and LB agar (Luqiao, Beijing, China) at 37 ℃ with shaking at 180 rpm for 12–24 h.

**Table 2:** Information of the samples collected for *Salmonella* strains isolates

Bacterial Strain^a^	Cities^b^	Serotype^c^	Separation date	Bacterial Strain^a^	Cities^b^	serotype^c^	Separation date
1	Weifang	ST	2019	14	Yantai	ST	2019
2	Weifang	SE	2019	15	Yantai	ST	2019
3	Weifang	SE	2019	16	Yantai	ST	2019
4	Liaocheng	SE	2019	17	Liaocheng	SP	2020
5	Liaocheng	SE	2019	18	Liaocheng	SP	2020
7	Liaocheng	SP	2019	20	Liaocheng	SP	2020
8	Dongying	SE	2019	21	Liaocheng	SP	2020
9	Yantai	SE	2019	22	Liaocheng	SP	2020
10	Yantai	ST	2019	23	Liaocheng	SE	2020
11	Yantai	ST	2019	24	Liaocheng	SE	2020
12	Yantai	ST	2019	25	Liaocheng	SE	2020
13	Yantai	ST	2019				

^a^ 1–25 represents the sample number of the strain

^b^ Names of cities where the serum samples were located in Shandong, China

^c^ *SE* stands for *Salmonella* Enterica, *ST* stands for *Salmonella* Typhimurium, *SP* stands for *Salmonella* Pullorum

### Phage isolation and purification

The phages were isolated from sewage pits and feces of seven broiler farms in different cities in Shandong Province. First, 5 mL or 5 g of the sample was mixed with 5 mL of 0.9% NaCl, vortexed to obtain a homogeneous mixture, and centrifuged (centrifuge H1650, Xiangyi, China) at 13,000 rpm for 5 min. The centrifuged supernatant was filtered through a 0.22 μm filter. Subsequently, about 500 µL of *Salmonella* (10^8^ CFU/mL) was cultured overnight and when the logarithmic phase of the bacteria is reached, it was mixed with 5 mL preheated semi-solid LB medium and spread on the LB agar plate (double-layer plate method). About 10 µL of the crude filtrate was added to the solidified semi-solid LB plate, cultured at 37 ℃ for 24 h. The plates were observed for the presence of transparent areas or plaques at the inoculation point.

Then, the transparent plaque was collected from each plate and inoculated into 1.5 mL centrifuge tube containing sodium chloride magnesium sulfate (SM) buffer (50 mM Tris–HCl, 100 mM NaCl, 10 mM MgSO_4_ [pH 7.5] [final concentration]) (Carnoss, China). After amplifying the phage in the solution at around 25 °C, the solution was centrifuged at 12,000 rpm for 10 min and the supernatant was finally collected by passing through a 0.22 µm filter. The filtered supernatant was titrated again by the double-layer plate method. After incubation at 37 °C for 24 h, the different plaques were selected based on the size and transparency of the plaques and resuspended in 100 µL SM buffer. The purification was continued 6 times until homogeneously-distributed plaques-containing phage isolates were obtained. The purified phage was stored in the precooled LB broth, mixed with 50% (v/v) glycerol, and stored in the refrigerator at -80 °C until further analysis.

### Plaquing efficiency of phage

The purified phage (100 µL) was activated using an LB broth (5 mL) at 37 °C, and the filtered supernatant was taken. The concentration of the PFU was calculated by counting the number of plaque using the double-layer plate method, and the initial concentration (PFU/mL) was calculated using the formula below:

$$The\;initial\;phage\;concentration\;(PFU/mL)\hspace{0.17em}=\hspace{0.17em}number\;of\;plaques\hspace{0.17em}\times\hspace{0.17em}5\hspace{0.17em}\times\hspace{0.17em}dilution\;factor$$

### Phage host range

The host range of the phages was determined by the Double-layer plate drip method using the strains shown in Table 2. Briefly, 10 μL of the purified phage culture medium was added dropwise to the LB plate which was covered with different serotypes of *Salmonella*. After culturing overnight, the spots were divided into three categories based on clarity of spots: clear, turbid, and unresponsive [14, 15]. The experiment was repeated three times to obtain reliable results.

### Thermal and pH stability analysis

The thermal stability was determined by culturing the isolated and purified phage samples at 40 ℃, 50 ℃, 60 ℃, 70 ℃, and 80 ℃, and the phage titer was determined by the double-layer agar method 20 min, 40 min, and 60 min after culture [15, 39, 46]. The pH stability was determined by mixing the isolated and purified phage samples in a series of test tubes containing SM buffer with different pH values (1–13, adjusted using NaOH or HCl solution). After culturing at 37 °C for 1 h, the phage titer was titrated by the double-layer agar method [14, 39, 46].

### Determining the optimal MOI

To test the best MOI, the host *Salmonella* was diluted to 1 × 10^8^ CFU/mL, and the phage solution was mixed with the diluted bacteria in the proportions of 0.1, 0.01, and 0.001 respectively, and then cultured for 3 h at 37 ℃. Then, the cultured mixture was centrifuged at 12,000 rpm for 10 min, filtering the supernatant through a 0.22 µm filter, and then, the titer of phage filtrate was determined by the double-layer plate method. The experiment was repeated three times, selecting the diluent producing the highest phage titer as the best MOI [39].

### One-step growth curve

A one-step growth curve assay was carried out based on a previously published method [47]. The bacterial culture of the host *Salmonella* was mixed with the phage lysate of optimization MOI and incubated for 5 min at 37 ℃. The mixture was then centrifuged at 12,000 rpm for 10 min, the supernatant was discarded, and the pellet was washed twice with LB broth. Subsequently, the pellet was resuspended in an equal volume of LB broth and incubated at 37 ℃ with constant agitation at 180 rpm. About 100 μL of the samples were collected every 10 min, for 120 min, and diluted in LB broth. The samples were then plated onto the agar plates using the agar overlay method [48], and the titer of phage filtrate was determined using the double-layer plate method. The time interval between the phage adsorption and the beginning of bacterial lysis was considered the latency period. To determine the burst size, the average number of final free phage particles was divided by the number of initial free phage particles. The experiment was repeated three times.

### Transmission electron microscopy

The purified phage with the best MOI was mixed with the host strain and cultured to the logarithmic phase. Then, the mixture was mixed with 5 mL preheated semi-solid LB agar and covered on the LB agar plate. After overnight culture at 37 ℃, 10 mL of the SM buffer was added to the culture plate and oscillated at 37 ℃ and 100 rpm for 4 h. After centrifuging the culture mixture for 5 min at 12,000 rpm, the supernatant was filtered through a 0.22 µM filter, and then the titer of the phage filtrate was determined by the double-layer plate method. The phage filtrate was negatively stained using 2% phosphotungstic acid (w/v, pH 7.0) and observed using a JEM-1200EXII electron microscope (JEOL, Japan). The micrographs were captured at an accelerating voltage of 80 kV the morphology of the phage was observed, and the length of the head and tail of the phage were measured by the ImageJ software [49].

### Nucleic acid isolation and categorization

The phages were concentrated before isolating the nucleic acids. Firstly, the free DNA and RNA in the phage samples were removed with RNase A and DNase I for obtaining a final concentration of 1 μg/mL phage, and cultured at 37 °C for 30 min. Next, NaCl was added to the phage mixture for obtaining a phage with a final concentration of 1 mol/L and cultured overnight at 4 ℃. After centrifuging the mixture at 12,000 rpm at 4 °C for 10 min, the supernatant was treated with PEG 8000 to obtain a mixture having a final concentration of 10% (w/v) and cultured overnight at 4 °C. The mixture was centrifuged at 12,000 rpm and 4 ℃ for 10 min. After discarding the supernatant, the precipitate was air-dried at around 25 °C, resuspended in the SM buffer, and cultured at 4 ℃ for 2 h to disperse the particles [14, 15]. The buffer was stored at − 20 °C until further use. The whole bacteriophage genome was extracted using the phenol–chloroform extraction method [50]. The whole bacteriophage genomic was then digested using the restriction enzymes DNase I (Hunan Accurate Biotechnology, China), RNase A (Hunan Accurate Biotechnology, China), and Mung Nuclease, respectively. The genotypes of the enzyme digested products were evaluated using agarose gel electrophoresis.

### Intestinal recovery rate of the feeding phage *in vivo*

In this study, 35 SPF chickens (Jinan Spafas Poultry Co., Ltd. Shandong Province, China) were randomly divided into the phage-fed groups (*n* = 30) and control groups (*n* = 5) on day 0. The feces of every chicken were identified as free of phages that had infected the host bacteria. On the 5th day, 100 µL of phage at 10^6^ PFU/mL were fed to the chickens in the phage-fed group, and every 3 chickens were euthanized at 5 min, 10 min, 15 min, 20 min, 25 min, 30 min, 60 min, 120 min after feeding, respectively, while the chickens in the control group were euthanized at the end of the experiment. The intestinal contents were collected at each time point both in the phage-fed as well as the control groups and mixed with 5 mL of 0.9% NaCl. Then, each sample was vortexed to obtain a uniform mixture and centrifuged at 13,000 rpm for 5 min. The supernatant was centrifuged and filtered through a 0.22 µm filter. Finally, the lysis behavior of phage filtrate and host bacteria was measured by the double-layer drip plate method, and the titer of phage was measured by the double-layer plate method.

### Recovery rate of the phage *in vitro*

For this study, 35 SPF chickens (Jinan Spafas Poultry Co., Ltd. Shandong Province, China) were subdivided into the phage mixing groups (*n* = 30) and control groups (*n* = 5) on day 0, and the feces of every chicken were identified as being free of phages infecting the host bacteria. On the 5th day, all the chickens were euthanized, and the intestinal contents and gastric juice were collected. The intestinal contents and gastric juice of the phage mixing group were mixed with 100 µL of phage at 10^6^ PFU/mL, while the intestinal juice and gastric juice mixture of every 3 chickens were collected at 5 min, 10 min, 15 min, 20 min, 25 min, 30 min, 60 min, 120 min after mixing respectively, and each sample was mixed with 5 mL 0.9% NaCl. The intestinal contents and gastric juice of the control group were combined with 100 µL of 0.9% NaCl and collected at 5 min after mixing. All the samples were centrifuged at 13,000 rpm for 5 min, and the supernatant was filtered through a 0.22 µm filter. Finally, the lysis behavior of phage filtrate and host bacteria was measured by the double-layer drip plate method, and the titer of phage was measured by the double-layer plate method.

### Antibacterial activity of the phage vB_SalP_LDW16 in the chicken model treated with *Salmonella* Enteritidis S64

To detect the antibacterial activity of the phage vB_SalP_LDW16, the experimental strain, *Salmonella* Enteritidis S64 (*S.* Enteritidis S64) was selected according to the clinical pathogenicity of the different serotypes of *Salmonella* stored in the laboratory, and then, the 50% lethal dose (LD50) of *Salmonella* Enteritidis S64 was determined. Firstly, 40 1-week-old SPF chickens were randomly divided into 4 groups in the isolators and each group was treated with intraperitoneal injections with different doses of *S.* Enteritidis S64 (10^7^, 10^8^, 10^9^, and 10^10^ CFU) [51]. The LD50 of *Salmonella* Enteritidis S64 was calculated and determined according to the mortality of each chicken group and used in the subsequent treatment trial.

The protective effect of the phage vB_SalP_LDW16 on the chicken was tested by randomly dividing 140 1-week-old SPF chickens into seven groups, namely blank (Normal saline), phage, and *Salmonella* controls, and phage, amoxicillin (Amx), florfenicol (FFC), and neomycin-treated groups. The chickens in each group were housed in isolators and those in groups 3–7 was intraperitoneally injected with with 10^8^ CFU/mL of *S.* Enteritidis S64, and those in groups 1–2 was intraperitoneally injected with an equal dose of normal saline. The water supply to each group was stopped 2 h before the treatment. Subsequently, 2 h after the bacterial challenge, groups 2 and 4 were orally administered with a single dose of 10^6^ PFU/mL of the phage vB_SalP_LDW16 according to relevant literature research [39, 46], while the groups 1 and 3 were fed the same dose of normal saline. Two hours after the bacterial challenge, groups 5–7 were orally administered with a certain dose of amoxicillin (1,122,102,077, Lukang Medicine, China), florfenicol (FB2012076, Jiangsu Hengsheng, China), and neomycin (202,006,222, Three Gorges in Yichang, China) as per the dose suggested in the relevant literature [52, 53]. The clinical symptoms and mortality of the chickens were observed every day for 7 consecutive days after treatment. At the same time, 3 chickens in each group were randomly selected at different time points after the treatment for blood collection to detect the dynamic changes in the bacteria in the chickens, and the feces were randomly collected from each group to detect the dynamic changes in the phages. After 7 days of treatment, the weight of the remaining chickens in each group was weighed and the pathological changes in the tissues and organs in each group were observed through pathological sections.

### Statistical analysis

The data were analyzed statistically using a one-way analysis of variance (ANOVA) of the statistical package SPSS 20.0 (SPSS Inc., Chicago., IL). All the data were represented as the mean ± standard deviation (SD) for at least 3 biological replicates under the same conditions. The *P*-values less than 0.05 were considered statistically significant (* *P* < 0.05, ** *P* < 0.01, and *** *P* < 0.001).

## Data Availability

The data supporting the findings are included in the manuscript.

## Abbreviations

MOI: Multiplicity of infection
PFU: Plaque-forming units
CFU: Colony-forming unit
SE: *Salmonella* Enterica
ST: *Salmonella* Typhimurium
SP: *Salmonella* Pullorum
CRISPR/Cas: Clustered regularly interspaced short palindromic repeats-associated nuclease system
LB: Luria–Bertani
SM: Sodium chloride magnesium sulfate
TEM: Transmission electron microscopy
Amx: Amoxicillin
FFC: Florfenicol
SD: Standard deviation
LD50: 50% Lethal dose
HE: Haematoxylin and eosin
